# Catastrophic and impoverishing out-of-pocket health expenditure in Ethiopia: evidence from the Ethiopia socioeconomic survey

**DOI:** 10.1186/s13561-025-00602-1

**Published:** 2025-03-01

**Authors:** Yamlak Bereket Tadiwos, Meseret Molla Kassahun, Anagaw Derseh Mebratie

**Affiliations:** Department of Health Systems Management and Health Policy, School of Public Health, College of Health Science, Addis Ababa University, Addis Ababa, Ethiopia

**Keywords:** Out-of-pocket expenditure, Catastrophic health expenditure, Impoverishing health expenditure

## Abstract

**Background:**

Out-of-pocket payment remains one of the ways to finance health care in Ethiopia accounting 31%. These out-of-pocket health expense leads citizens’ face catastrophic and impoverishing expenditure. The most recent survey-based study of catastrophic and impoverishing health expenditure was done from the 2015/16 consumption and expenditure survey with finding of 2.1% and 1% respectively.

**Objective:**

To assess catastrophic and impoverishing out-of-pocket health expenditure and the determinant factors of catastrophic health expenditure in Ethiopia, 2023 from the 2018/19 socioeconomic survey.

**Methodology:**

A secondary data from Ethiopian socioeconomic survey 2018/19 conducted by Ethiopia’s Central Statistical Agency and World Bank was used to assess the catastrophic and impoverishing health expenditure at the national and subnational level by the Wagstaff and Van Doorslaer and Xu et al. methodology. Then binary logistic regression was computed by the STATA (ver.12) software to assess the determinant factors of catastrophic health expenditure.

**Result:**

From 6770 households 1.49% and 0.89% of them in Ethiopia faced catastrophic and impoverishing health expenditure respectively at 10% threshold level and households having a member with more facility visit had increased likelihood of facing catastrophic health expenditure (AOR = 2.45, 95%CI; 1.6—3.8) and also having member being hospitalized in the household had increased odds of facing catastrophic health expenditure (Adjusted odds ratio, AOR = 1.9, 95% confidence interval, CI; 1.19- 3.16). On the contrary, there is a decreased likelihood of facing catastrophic health expenditure among those who were insured for health (AOR = 0.58, 95%CI; 0.35- 0.97) and was in the richest consumption quintile group (AOR = 0.6, 95%CI; 0.47- 0.65).

**Conclusion and recommendation:**

The finding indicates that there are still notable households facing catastrophic and impoverishing out-of-pocket health expenditure in Ethiopia especially in the lower consumption quintiles indicating inequity. In addition it is found that those with health insurance coverage, lower hospitalization and health service utilization had lower chance of facing catastrophic health payment. So it is suggested that activities that reduce hospitalization rate, increase insurance coverage and addressing the poor must be in place so that the catastrophic health cost incurred can be lowered at national level.

## Introduction

All member countries of the United Nations (UN) aim to achieve sustainable development goal(SDG) by 2030 encompassing the goal of attaining universal coverage through financial risk protection, access to essential quality health service and access to safe, effective, quality and affordable essential medicines and vaccines for all. One factor that hinders financial risk protection in a country is out-of-pocket(OOP) payments for obtaining health services which remains one of the ways to finance health expenditure and leads households and individuals face financial catastrophic expense or impoverishment [1, 2]. Health expenditure is defined as catastrophic if it is above a certain agreed fraction of household’s income or total expenditure [3]. If the expense gets up to the level where it pushes the individual or household below the agreed international or national poverty line it is said to be impoverishing health expenditure [4].

Even though out-of-pocket health payment is known to be the most regressive method of funding for healthcare and in spite that guaranteeing access to health services for all without facing financial hardship is mandatory for sustainable development and economic growth millions of people seeking care around the world are still struggling financially due to the burden of out-of-pocket medical expenses [2, 5].

In addition access to healthcare is hampered by out-of-pocket (OOP) health costs and according to various studies, even minor OOP expenses can put patients and their families in poverty which in return place low-income communities at significant financial risk and prohibit their right of getting adequate health service [6].

Globally, about 930 million people (12.7%) faced catastrophic health expenditure (CHE) by sacrificing at least 10% of their household budgets paying for health care out of their own pockets, and about 90 million people (1.2%) are still being pushed in to extreme poverty (IHE) as a result of out-of-pocket payment for healthcare [7] which may lead people not to access the health service due to lack of financial capability, especially for the poor and cut back their basic necessities unnecessarily respectively [8, 9].

In low-income countries, out-of-pocket payments account for 30% of total funding [10]. In addition, in these countries average household out-of-pocket healthcare expenditure per individual has risen by 66% between 2000 and 2017 [11].

In developing countries, including Ethiopia, the payment method for the healthcare cost is mainly OOP paid at the time of seeking care or at the point of service delivery, which could impede healthcare access or otherwise lead to impoverishment due to healthcare utilization [12].

From the Ethiopia's seventh national health account report of 2016/17, OOP payment contributes for about 31% of the overall funding, or 1.3 percent of the country’s gross domestic product (GDP), considerably higher than the global recommended target of 20% and as a percentage of total private health expenditure, out-of-pocket (OOP) spending exceeds 93% [13].

In Ethiopia, not only did the rate of catastrophic rate increased over time from 2% in 2011 to 5% in 2015, but the poorest wealth quintile went from a catastrophic rate of 3.4% in 2011 to 9% in 2015, an increase of almost 6 percent [14]. But according to a recent study estimated from national representative data it was found to be 2.1% CHE incidence and 0.8% IHE. High rates of CHE and IHE was recorded in Afar (5.8%, 5%) [15]. The various findings are due to the difference in time period of the studies.

Regarding factors that are determinants of catastrophic health expenditure it was found in many national studies that inpatient care, location of the household, utilization of private health facilities, health insurance coverage, utilization of health care service, hospitalizations, wealth quintile and presence of elderly and children in the household are significant determinants [5, 16–20].

### Rationale of the study

Despite global progress in achieving Universal Health Coverage (UHC), millions of households in low- and middle-income countries (LMICs) remain at risk of financial hardship due to out-of-pocket (OOP) payments for healthcare. In Ethiopia, OOP payments account for approximately 31% of total health expenditure, far exceeding the globally recommended target of 20%, and remain a major barrier to equitable healthcare access. However, national estimates of catastrophic health expenditure (CHE) and impoverishing health expenditure (IHE) are outdated, limited, or inconsistent, necessitating a fresh and comprehensive analysis. This study is critical for several reasons:

1. Addressing data gaps in financial protection

The most recent study of CHE and IHE in Ethiopia, based on 2015/16 data, reported incidences of 2.1% and 1.0%, respectively. However, substantial socioeconomic changes, health financing reforms, and programmatic interventions (e.g., expansion of community-based health insurance [CBHI]) have occurred since then. Updated evidence is essential to assess the impact of these reforms and identify areas where progress has stagnated or declined.

2. Regional disparities and inequities

Previous studies have highlighted significant regional disparities in CHE and IHE in Ethiopia, with certain regions disproportionately burdened by high OOP payments. Yet, little is known about how these disparities have evolved over time. This study fills this gap by providing updated national and subnational estimates, allowing policymakers to target interventions in regions with the greatest need.

3. Limited understanding of determinants

While global studies have identified common determinants of CHE, such as hospitalization, health insurance coverage, and socioeconomic status, the drivers of CHE in Ethiopia’s unique context remain underexplored. By analyzing the determinants of CHE, this study provides actionable insights to inform tailored policy solutions that address the root causes of financial hardship.

4. Monitoring progress toward UHC

Achieving UHC is a cornerstone of the Sustainable Development Goals (SDGs), requiring all member states, including Ethiopia, to ensure financial risk protection and equitable access to healthcare. This study serves as a critical benchmark for monitoring Ethiopia’s progress toward UHC and assessing the effectiveness of health financing policies and programs.

5. Advocacy for the poorest and most vulnerable

The findings of this study are particularly important for advocating for the poorest households, who are disproportionately affected by financial hardship. By quantifying the intensity (mean positive overshoot) and incidence of CHE and IHE, this study highlights the depth of inequality and makes a compelling case for scaling up pro-poor health financing reforms.

6. Contribution to global literature

Ethiopia represents a compelling case for studying CHE and IHE due to its low-income status, heavy reliance on OOP payments, and recent health financing reforms. This study contributes to the global discourse on financial risk protection by offering lessons that can be applied to other LMICs with similar socioeconomic and healthcare challenges.

### Policy relevance and implications

This study provides policymakers and stakeholders with evidence-based insights to:

- Scale up CBHI enrollment and address barriers to its adoption, particularly in rural areas.
- Strengthen fee waiver and exemption programs to reduce the financial burden on the poorest households.
- Invest in preventive and primary healthcare services to reduce costly hospitalizations.
- Allocate resources equitably to address regional disparities in CHE and IHE.

## Methodology

### Data source

A secondary data from Ethiopian socioeconomic survey 2018/19 conducted by Ethiopia’s Central Statistical Agency and World Bank was used to assess the catastrophic and impoverishing health expenditure at the national and subnational level by the Wagstaff and van Doorslaer and Xu et al. Methodology [21]. Then binary logistic regression was computed by the STATA (ver.12) software to assess the determinant factors of catastrophic health expenditure.

### Data processing and analysis techniques

After data acquisition from the World Bank micro data website in excel format it was exported in to STATA format and was labelled for each variable. Then it was assessed and cleaned for inaccuracies. Since the unit of analysis used was household the socio-demographic, health and health cost data were collapsed with mean and median values then merged.

Following this descriptive statistic method of analysis (i.e. frequency, mean, median etc.) was computed to explain the socio-demographic, health and health related cost variables. Then the catastrophic and impoverishing health expenditure was calculated by the wagstaff and vandoorslaer and Xu et al. methodology. Then after, bivariate analysis was computed to determine the significance level of association of factors found to be determinants in different literatures.

Following this binary logistic regression was computed to assess the determinant factors of catastrophic health expenditure (prob > chi2). At last the finding has been presented using tables, bar graphs, and pie charts accordingly.

### Measurement of outcomes

Overall five aspects of catastrophic and impoverishing expenditure were analyzed in this paper: (I) incidence of catastrophic health expenditure (II) intensity of catastrophic health expenditure, (III) poverty headcount (IV) poverty gap and (V) determinants of catastrophic health expenditure.

#### Incidence and intensity of catastrophic health expenditure

##### Wagstaff and van Doorslaer methodology

The incidence of CHE indicates the percentage of households that faces catastrophic health expenditure. It is calculated using the catastrophic payment headcount which is the proportion of OOP healthcare expenses that surpass a certain threshold in relation to the household consumption expenditure [22]

$$H=\frac{1}{N} \sum CHEi$$

where, H = catastrophic headcount.

N = sample size

$$\text{CHE}=\frac{Ti}{Xi}$$

where, CHE = catastrophic health expenditure.

T_*i*_ = OOP expenditure for health.

X_*i*_ = Household consumption expenditure or household non-food expenditure.

CHE is defined when the above ratio is greater than the chosen cutoff threshold which might vary according to country’s context (5%, 10%, 15%, 20%, 25%, and 40% of both total consumption expenditure and non-food expenditure). However the incidence does not indicate by how much percentage it is causing catastrophe so the intensity of catastrophic expenditure (catastrophic expenditure overshoot) can be calculated by the formula shown below

$$Oi= CHEi\{\left(\frac{Ti}{Xi}\right)-Z\}$$

where, O_i_ = catastrophic expenditure overshoot.

T_*i*_ = OOP expenditure for health.

X_*i*_ = Household consumption expenditure or household non-food expenditure.

Z = threshold budget share.

The threshold value represents the point at which household OOP expenditures can impose a severe disruption to basic living conditions and the specific threshold value can vary when the denominator for calculating the head count is either total income/expenditures or capacity to pay.

The measurement that relates the incidence and intensity of CHE is the mean positive overshoot (MPO). It calculates the mean percentage of OOP health expenditure that surpasses the threshold among households incurring CHE.

$$MPOi =\frac{Oi}{Hi}$$

##### Xu et al. Methodology

This methodology defines catastrophic expenditure from capacity to pay. Health expenditure is seen as catastrophic if the total health expenditure of a household equals or greater than 40% of the total household’s capacity to pay [23].

Capacity to pay is calculated using consecutive steps. First equivalent household size will be determined by calculating the power of the actual household size with $\beta$ which is obtained from regression equation ($\beta$ =0.56).

$$eqhhsize= {HHsize}^{\beta }$$

Then equivalent food expenditure is calculated by dividing food expenditure for equivalent household size: $eqfoodexp$= $\frac{foodexp}{eqhhsize}$

Then the food expenditure share (*foodexp*ℎ) for each household will be determined by dividing the food expenditure with total household expenditure.

$$foodexph= \frac{foodexp}{totHHexp}$$

Then the household food expenditure as share of total household expenditure in between 45 and 55th percentile across the whole sample will be determined. This percentile range defines the poverty line.

Next household subsistence expense (*SEi*) is calculated by multiplying the equivalent household size (*eq*ℎℎ*size*) by the poverty line (Pl*ine*)

$$SEi=eqhhsize * Pline$$

Finally household capacity to pay (*CTPi*) is calculated as;

$$CTPi = TotalHHexp - SEi when SEi \le foodexph\text{ Or}$$

$$CTPi = TotalHHexp - foodexph when SEi> foodexph$$

#### Impoverishing health expenditure

##### Wagstaff and Van Doorslaer methodology

Impoverishing health expenditure is measured by poverty headcount (denoted Hp) and poverty gap (Gp). Hp determines the number of households living below the poverty line as a proportion of all households, and the poverty intensity (Gp) measures by how much a household is far below the poverty line.

$$Hp=\frac{1}{N}*\sum Bi$$

where Bi is defined when OOP health expenditure (Ei) < poverty line.

$$Gp=\frac{1}{N}* \sum Di$$

Where

$$Di= Ei- Pline$$

Normalized mean positive Gp (NmGp) can be calculated as the average poverty gap of the poor divided by the poverty headcount*:* $NmGp =\frac{Gp}{Hp}$

#### Determinants of catastrophic health expenditure

To assess the determinant factors of the incidence of CHE multivariable binary logistic regression method of analysis was computed after checking for assumptions of logistic variables mentioned above. Then results with p value < 0.05 and odd ratio other than 0 values were taken to conclude the study.

### Operational definition

Out-of-pocket expenditure: Expense incurred by a household or individual at the point of service utilization provided by health professional without reimbursement from a third party.

Catastrophic health expenditure: High amount of out-of-pocket expense incurred for health as a share of household income which is greater than specific threshold of non-food or total expenditure, and capacity to pay.

Impoverishing health expenditure: Out-of-pocket health expenditure that leads a household living standard below national or international poverty line.

Capacity to pay: The amount that is defined from household after subtracting the expenses on basic needs from the total household consumption expenditure.

Non-food expenditure: Expenses spent on goods and services other than food.

### Ethical consideration

Since all the data was available online there was no need to obtain permission letter but the data was accessed after filling the access agreement form which obliges to cite the data owner (i.e. Central Statistical Agency) and to submit copy of the finding of the study to the Central Statistical Agency and the Development Data Group Division of the World Bank [21].

## Result

### Socio-Demographic and economic characteristics

The survey was conducted from 6770 households having 29,503 individuals with 14,319(48.5%) males and 15,184(51.5%) females in all the 9 regions. Those under the age of 15, between 15 and 64 and above 64 were 11,940(40.47%), 16,584(56.2%) and 979(3.32%) respectively.

The average household size at national level was found to be 4.24 with Somalia region taking the lead by mean household size of 5.69 followed by SNNP (4.57) and Gambella (5.47). 54% of the HHs (3,655) lived in the urban areas whereas the remaining 46%( 3,115) lived in the rural areas.

Regarding their marital status 45.4% of the individuals were single and 45.3% of them were married, from these number of households 1,594(54.2%) of them were illiterate and employment status for 12 month preceding the survey was 1.5% (Table 1).

**Table 1:** Surveyed Household characteristics across each region of Ethiopia 2018/19

Region	Av HH size	Number by sex (%) Male Female		Rural/urban HHs (%)		Literacy	Employ-ment status (%)
Tigray	3.79	1309 (47.3)	1457 (52.7)	393 (58.2)	283 (41.9)	1468 (61.2)	1.49
Afar	4.13	1169 (50.8)	1133 (49.2)	299 (46.4)	225 (53.6)	737 (39.2)	1.02
Amhara	3.81	1447 (48.1)	1561 (51.9)	479 (63.9)	271 (36.1)	1300 (50.7)	1.15
Oromia	4.45	1705 (49)	1778 (51)	453 (60.2)	300 (39.8)	1475 (49.7)	1.86
Somalia	5.69	1772 (50.7)	1723 (49.3)	355 (58.2)	255 (41.8)	1237 (39.3)	0.64
Benishangul gumuz	4.03	760 (50.3)	751 (49.7)	169 (46.4)	195 (53.6)	748 (57.7)	1.4
SNNP	4.57	1594 (48.3)	1704 (51.7)	422 (61.1)	269 (38.9)	1517 ((53.2)	0.56
Gambella	4.47	1127 (49.9)	1133 (50.1)	195 (39.4)	300 (60.6)	1306 (66.6)	0.57
Harar	3.76	1010 (48)	1093 (52)	190 (42.2)	360 (57.8)	1193 (64.9)	1.18
Addis Ababa	3.79	1311 (44.4)	1645 (55.6)	0	778 (100)	2393 ((88.4)	5.7
Dire Dawa	3.96	1115 (48)	1206 (52)	160 (27.6)	419 (72.4)	1422 (68.5)	1.5
National	4.24	14,319 (48.5)	15,184 (51.5)	3115 (46)	3655 (54)	14,796 (57.6)	1.5

**Fig. 1:**
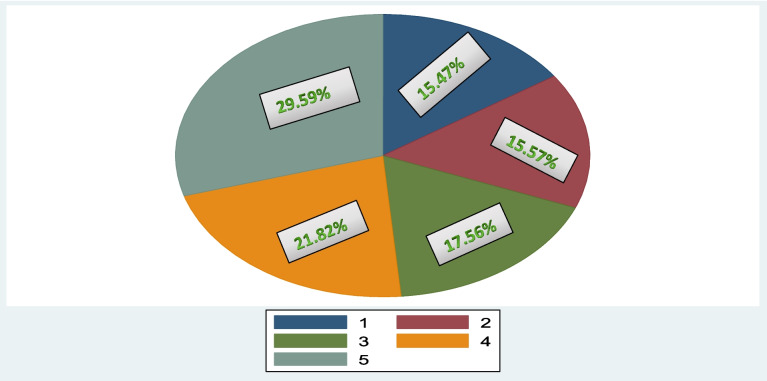
Consumption quintile of surveyed households in Ethiopia 2018/19

Based on their real per adult equivalent total consumption, households were sorted in increasing order and grouped in to 5 quintiles, with quintile 1 and 5 depicting the poorest and richest 20% households respectively (Fig. 1).

### Health status and health related costs

From all the households 2,727(40.3%) of them reported at least one member have been sick 1 month preceding the survey with Tigray (52.9%) taking the leading percentage followed by Amhara (50.5%). From these households who needed an appropriate remedy for their sicknesses were 2,484(91.1%) households.

When coming to computing the mean national total consumption per adult equivalent it was found to be 21,880 ETB whereas the Mean OOP health payment per adult equivalent and Share of OOP payments to total consumption were 130.10 ETB and 0.89% from the total cost respectively (Table 2).

**Table 2:** Self-reported illness, health seeking behavior and consumption of surveyed households across each region of Ethiopia 2018/19

Region	Self-reported illness by HH	Health seeking behavior	Mean total consumption per adult equivalent (ETB)	Mean OOP per adult equivalent (ETB)	Share of OOP payments to total consumption %
Tigray	226 (52.9%)	197 (87.2%)	20,616.998	88.47	0.46%
Afar	243 (46.4%)	229 (94.2%)	21,516.246	93.68	0.58%
Amhara	379 (50.5%)	336 (88.6%)	16,003.2	94.57	0.85%
Oromia	328 (43.6%)	311 (94.8%)	18,366.73	106.7	0.67%
Somalia	185 (30.3%)	195 (> 100%)	18,230.528	109.75	1.1%
Benishangul gumuz	118 (32.4%)	197 (> 100%)	20,904.39	438.31	2.28%
SNNP	209 (30.3%)	211(> 100%)	17,718.683	175.74	1.97%
Gambella	262 (52.9%)	216 (82.4%)	21,482.659	174.36	1.13%
Harar	225 (40.9%)	188 (83.5%)	28,739.535	78.67	0.46%
Addis Ababa	347 (44.6%)	280 (80.7%)	31,491.207	106.08	0.46%
Dire Dawa	205 (35.4%)	197 (96.1%)	26,204.927	104.63	0.49%
National	2727(40.3%)	2484(91.1%)	21,880.51	130.1	0.89%

Self-reported illness and health seeking behavior was found to be higher among the middle consumption quintiles. In addition the mean total consumption and mean OOP health payment per adult equivalent was increasing as one goes from the lowest consumption quintile to the highest one. On the contrary share of OOP health payments to total consumption was higher among the poorest consumption quintiles (Table 3).

**Table 3:** Self-reported illness, health seeking behavior and consumption of surveyed households across their consumption quintiles 2018/19

Consumption quintile	Self-reported illness of at least one member in the household (%)	Health seeking behavior of at least one member in the household (%)	Mean total consumption per adult equivalent (ETB)	Mean OOP per adult equivalent (ETB)	Share of OOP payments to total consumption %
1	421 (40.2%)	387 (36.9%)	5036.39	84.76	1.86%
2	417 (39.6%)	396 (37.6%)	9074.56	105.31	1.20%
3	478 (40.2%)	449 (37.8%)	13,235.26	98.28	0.77%
4	611 (41.4%)	577 (39.1%)	19,582.7	138.46	0.72%
5	800 (39.9%)	724 (36.2%)	44,250.13	179.57	0.46%

From all households the national percentage of the dependent age groups was 36.9% with Addis Ababa having the largest percentage (57.19%). The annual percent of hospitalization of at least one person in the household was 49.87% and it is highest in the Amhara region with 57.86 percentages.

From those seeking care the ones who got health service from private and non-governmental facilities were 30.47% of the households and proportion of households having health insurance coverage was 26.08 with Harar being the leading region with 54.54 proportions of households (Table 4).

**Table 4:** Health related variables to assess catastrophic health expenditure of surveyed households across each region 2018/19

Region	Presence of elderly(> 65 years) and child(< 5 years) in the HH %	Hospitalization of at least one HH member %	Presence of utilization of private and non-governmental health facilities %	Proportion of Health insurance coverage
Tigray	36.39%	44.53%	22.33%	41.42
Afar	55.15%	46.56%	36.83%	26.72
Amhara	50.4%	57.86%	36.13%	34.26
Oromia	53.78%	51.39%	36.25%	17.66
Somalia	44.42%	45.41%	27.05%	17.05
Benishangul gumuz	39.29%	53.85%	30.49%	16.48
SNNP	45.15%	46.70%	26.48%	10.28
Gambella	54.34%	57.78%	38.98%	17.17
Harar	48.54%	49.81%	25.81%	54.54
Addis Ababa	57.19%	53.98%	30.21%	33.67
Dire Dawa	52.85%	40.41%	25.22%	12.78
National	36.39%	49.87%	30.47%	26.08

### Catastrophic health expenditure

#### Incidence and intensity of catastrophic health expenditure

National catastrophic health expenditure using Wagstaff and Van Doorslaer Methodology by taking 10% threshold as a share of total household consumption expenditure was 1.49% with Benshangul-Gumuz, SNNP and Amhara were the top three regions with 4.12, 3.76, and 1.73 percent. The mean intensity of national catastrophic consumption expenditure or overshoot value was 20.36%. Regions with higher intensity were Gambella, Somalia and Dire-Dawa. In addition the mean percentage of OOP health expenditure that surpasses the threshold among households incurring CHE was 46.68.

Meanwhile computing catastrophic health expenditure as a share of non-food expenditure using 40% threshold was 4.69% and the average value of overshoot was 2.29% and the mean positive overshoot value was 53.86.

Other than the above one xu et al. method was applied to determine the catastrophic health expenditure by determining the capacity to pay level of each household and it was found that 2.48% of the households incur catastrophic health expenditure (Table 5).

**Table 5:** Incidence, intensity and mean positive overshoot of health consumption expenditure of surveyed households across each region of Ethiopia 2018/19

Region	CHE incidence (as a share of total consumption)%	Mean overshoot%	Mean positive overshoot	CHE incidence(as a share of non-food consumption expenditure)%	Mean overshoot per thousand	Mean positive overshoot	CHE using capacity to pay %
Tigray	0.74%	4.22%	27.04	1.92%	8.51	48.36	1.78%
Afar	1.15%	9.58%	44.99	3.43%	11.57	57.48	1.53%
Amhara	1.73%	10.76%	37.65	5.91%	30.73	51.81	3.46%
Oromia	1.06%	6.59%	35.15	5.44%	7.18	43.59	1.59%
Somalia	1.64%	30.17%	41.84	4.92%	19.69	52.29	2.29%
Benishangul gumuz	4.12%	23.46%	56.68	15.93%	49.58	61.22	6.32%
SNNP	3.76%	26.48%	52.52	7.09%	17.7	56.88	5.49%
Gambella	1.41%	30.32%	56.95	4.47%	20.64	55.19	3.23%
Harar	0.90%	11.66%	35.2	3.27%	10.68	57.89	0.36%
Addis Ababa	0.38%	27.86%	50.32	1.67%	7.03	41.77	1.67%
Dire Dawa	0.52%	29.53%	59.56	1.9%	20.7	57.92	0.69%
National	1.49%	20.36%	46.68	4.69%	22.92	53.86	2.48%

**Fig. 2:**
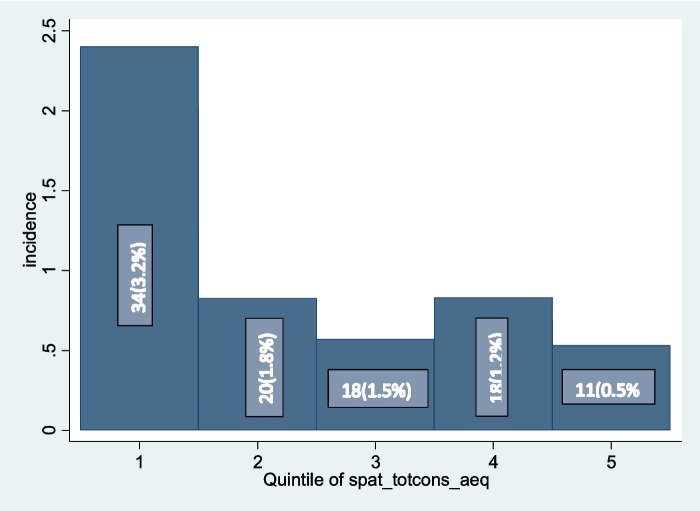
Incidence of CHE among each consumption quintiles of surveyed households in Ethiopia 2018/19

Incidence of CHE was found to be higher among households with lower consumption when using the total health consumption expenditure using 10% threshold. The incidence seems to decrease as one goes from the lowest to the highest consumption quintile level (Fig. 2).

#### Impoverishing health expenditure

Following the steps listed below (Xu et al. methodology) the poverty line was determined to be 7,338 ETB. After this households who were impoverished due to OOP health expenditure were 0.83% of the whole population with SNNP being the 1st than the rest with 1.88%. The intensity of impoverishment level was on average 2,490 ETB from the poverty line. The normalized mean positive intensity value was 1.46 (Table 6).

**Table 6:** Poverty headcount and intensity of poverty due to health expenditure of surveyed households across each region in Ethiopia 2018/19

Region	Poverty headcount (Hp) %	Poverty intensity (Gp) ETB	Normalized mean positive Gp (NmGp)
Tigray	0.74%	3419.76	1.46
Afar	0.38%	3409.5	0.94
Amhara	0.02%	1950.49	0.49
Oromia	0.27%	2104.63	0.48
Somalia	0.16%	2282.79	0.45
Benishangul gumuz	0.82%	686.67	0.11
SNNP	1.88%	2570.06	0.91
Gambella	1.01%	2687.99	0.85
Harar	1.09%	3456.45	3.81
Addis Ababa	0.13%	1210	0.19
Dire Dawa	0.52%	2963.66	0.71
National	0.83%	2489.59	1.46

When IHE was assessed among each consumption quintiles, only the 2 with lowest consumption quintiles incur impoverishing health expense whereas the richest 3 do not have health expense that leads them to become impoverished (Table 7).

**Table 7:** Impoverishing health expenditure among each consumption quintile in Ethiopia

Consumption quintiles	Impoverishing health expenditure	
	Number	Percentage
1	44	4.2
2	12	1.14
3–5	0	0

#### Determinants of catastrophic health expenditure

First bivariate analysis was computed for the different socio-demographic, socioeconomic and other variables to determine those to be computed with binary multivariable logistic analysis then household residence, health service utilization, hospitalization, private facility use, being insured for health and the consumption quintile group of the household had significant association with CHE ( *p* < 0.05).

After this binary multivariable logistic regression was done to determine which factors were associated with incurring catastrophic health expenditure at a share of total household expenditure with 10% cut off point.

Households who had more facility visit (Adjusted odds ratio, AOR = 2.45,95% confidence interval, CI;1.6- 3.8) had increased odds of facing CHE as compared to those having less facility visit and also having member being hospitalized (AOR = 1.9,95%CI;1.19- 3.16) had increased odds of facing CHE. Whereas, there is a decreased likelihood of facing CHE among those who were insured for health (AOR = 0.58, 95%CI; 0.35- 0.97) and those who are in the richest consumption quintile group (AOR = 0.6, 95%CI; 0.47- 0.65) (Table 8).

**Table 8:** Binary multivariable logistic regression of CHE as a share of 10% of total household expenditure 2018/19

Variables		CHE as a share of 10% of total household expenditure	
		Number	AOR(95% CI)
Health service utilization	Yes	2484 (91.1%)	2.45 (1.56, 3.84)
	No	243(8.9%)	
Hospitalization	Yes	337,650.13%))	1.9 (1.19, 3.16)
	No	2994 (49.87%)	
Health insurance coverage	No	5004 (73.92%)	5.8* (3.53*, 9.7*)
	Yes	1766 (26.08%)	
Consumption quintiles	Q1	1	
	Q2	2	0.362* (0.101*-1.29*)
	Q3	3	0.801* (0.291*-2.20*)
	Q4	4	0.62* (0.199*-1.539*)
	Q5	5	5.5* (4.7*,6.5*)

## Discussion

This study provides updated insights into catastrophic and impoverishing health expenditures (CHE and IHE) in Ethiopia, shedding light on both their prevalence and determinants using nationally representative data. The findings indicate that 1.49% of households face CHE and 0.83% experience IHE at the 10% threshold, with significant regional disparities observed. Regions such as Benishangul-Gumuz (4.12%), SNNP (3.76%) and Amhara (1.73%) reported the highest incidences, highlighting persistent inequalities in financial risk protection within the Ethiopian health system.

The CHE incidence observed in this study is lower than reported in other low- and middle-income countries, such as India (16.51%), Bangladesh (24.6%) and Kenya (11.7%) [16–18]. This discrepancy may reflect the positive impact of Ethiopia’s health financing reforms, including the expansion of community-based health insurance (CBHI) schemes and fee waiver programs. However, the findings align closely with prior Ethiopian studies, such as the 2015/16 survey, which reported CHE and IHE incidences of 2% and 1%, respectively. The small reduction over time may be attributable to the exclusion of households unable to afford care, which remains a critical limitation of studies relying on service utilization data.

The study underscores the protective role of health insurance against financial hardship, as households with insurance were significantly less likely to experience CHE (AOR = 0.58). However, with only 26.08% of households covered by health insurance, significant gaps remain in achieving universal coverage. Similarly, households in the richest quintile demonstrated a lower likelihood of CHE (AOR = 0.6), further highlighting the inequities in Ethiopia’s health financing system. Addressing these gaps requires scaling up insurance coverage, particularly in rural and low-income populations, and improving access to affordable health services.

The high odds of CHE among households with frequent facility visits (AOR = 2.45) or hospitalizations (AOR = 1.9) emphasize the need to strengthen preventive and primary healthcare services to reduce the burden of expensive secondary and tertiary care. Moreover, targeting regions with disproportionately high CHE, such as Benishangul-Gumuz and SNNP, should be a priority for policymakers to ensure equitable resource allocation.

Regarding the determinant factors of catastrophic health expenditure there is different results compared to findings in South Korea, America and China in which old age and place of residence were the determinant factors [24, 25]. These results might be attributed to the difference in that these countries are in the category of high income countries resulting in different socio economic condition hence having different factors leading to catastrophic health payment.

Whereas there are similar studies done using national survey (i.e. India, Zimbabwe, and Kenya) in which the determinant factors were health insurance coverage, consumption quintile, hospitalization and health service utilization rate in which those covered under health insurance scheme and households categorized under the richest consumption quintile had lower likelihood of facing catastrophic health payment while those who have member being hospitalized and increased rate of health service use had increased odd of facing catastrophic health expense [16–18, 20]. These findings might be due to similarities in the socio economic conditions (i.e. low and middle low income countries) in these countries and simultaneous activities done in these countries to achieve the goal of moving towards universal health coverage [16].

Regarding the poorer being the one to face the catastrophic health payment in Ethiopia, when CHE was assessed among each consumption quintile it was found that both were incurred in higher amount among the lower consumption quintiles than the rich consumption quintiles. Also findings in countries like India, Zimbabwe and Kenya have similar results showing that the poor is more inclined to face catastrophic and impoverishing payment for health than the rich ones [17, 20, 26]. These similarities are attributed to being in similar socio-economic state.

And in comparison to Ethiopian studies it is different finding relative to previous study done using the 2015/2016 ICHE survey which shows that the rich is more inclined to face catastrophic and impoverishing health expenditure but similar with the meta analysis finding with AOR = 3.09 (95% CI: 1.63, 5.86) *p*-value < 0.001 [15, 27]. These variations might be because of the time and place variation of the studies.

So the finding of this study indicates that in spite of the efforts made to reduce OOP payment for health by providing fee waiver and exemption service to the poor there is still inequality issue not being addressed especially in regions like SNNP and that this service needs to expanded in inadequate manner to the vulnerable society so that the country’s aim of achieving UHC can be realistic one [5].

Broader context: The findings contribute to the global discourse on achieving Universal Health Coverage (UHC). They highlight Ethiopia’s progress and ongoing challenges in reducing financial barriers to healthcare access. The regional disparities in CHE and IHE suggest that achieving UHC requires a context-specific approach, with tailored strategies to address unique socioeconomic and healthcare access barriers across different regions.

Potential interest of results: The results of this study have significant implications for both research and policy. By identifying specific determinants of CHE, this study provides actionable evidence for targeted interventions, such as expanding insurance coverage, subsidizing care for the poor, and improving access to preventive services. The findings also serve as a benchmark for monitoring Ethiopia’s progress toward UHC, offering a foundation for future studies to evaluate the long-term impacts of health financing reforms.

Furthermore, the study’s use of two complementary methodologies (Wagstaff and Van Doorslaer, and Xu et al.) enhances the robustness of its findings and sets an example for similar analyses in other low-income settings. This methodological rigor ensures that the results are both credible and relevant to international researchers and policymakers working on financial risk protection.

### Methodological discussion

#### Control over methods

In this study, we employed secondary data analysis using the 2018/19 Ethiopian Socioeconomic Survey conducted by the Central Statistical Agency and the World Bank. The methodology was carefully chosen to ensure rigor and replicability. We controlled the process through:

- Data Cleaning and Preparation: Ensuring accuracy by systematically labeling and merging variables.
- Statistical Rigor: The use of binary logistic regression and Wagstaff & van Doorslaer, Xu et al. methodologies ensured precise estimates of catastrophic and impoverishing health expenditures at both national and regional levels.
- Threshold Selection: We used multiple thresholds (10%, 40%, and capacity to pay) to assess robustness.
- Comparative Approach: Findings were compared across consumption quintiles and regions to highlight disparities and validate interpretations.

#### What could have been done?

While the chosen methods were appropriate for the research objectives, alternative approaches could have provided additional insights:

1. Longitudinal Analysis: Instead of a cross-sectional study, tracking households over multiple years could help assess trends in catastrophic and impoverishing expenditures more dynamically.
2. Mixed Methods Approach: Integrating qualitative interviews could have provided a deeper understanding of coping mechanisms among households facing financial hardship.
3. Inclusion of Indirect Costs: The study mainly focused on direct medical costs; incorporating transportation, lost wages, and informal payments would have captured a fuller picture of financial burden.
4. Geospatial Analysis: Mapping catastrophic expenditure rates by region or urban–rural divisions could enhance policy recommendations for targeted interventions.

#### Merits of the current methodology

- National Representativeness: The dataset is from a large, nationally representative survey**,** ensuring broad generalizability.
- Robust Analytical Framework: The use of Wagstaff & van Doorslaer, and Xu et al. methodologies ensures internationally recognized comparability.
- Policy Relevance: The study directly informs Ethiopian policymakers about regional disparities in health expenditure burdens.
- Efficiency & Cost-Effectiveness: Secondary data analysis eliminates the cost and time constraints of primary data collection while ensuring high-quality data.

### Limitations of the study

- Lack of primary data control: As a secondary data study, variables were predefined, limiting the ability to include additional indicators such as household debt, social support mechanisms, or perceived financial distress**.**
- Underestimation of burden: Households that avoided healthcare due to financial constraints were not captured, potentially underestimating catastrophic health expenditure.
- Cross-Sectional Design: The study cannot establish causality**,** only associations.
- Exclusion of Informal Healthcare Costs: Many Ethiopian households rely on traditional healers or informal care; these were not included in the analysis.

### Recommendation for future studies

- ✔ Broader Scope of Expenditure Analysis

Future studies should include both direct and indirect costs of healthcare. This study focused only on direct medical expenses, but indirect costs such as transportation, lost income, and caregiver expenses significantly contribute to the financial burden on households.

- ✔ Incorporating Households Excluded from Healthcare Utilization

many households may not seek healthcare due to unaffordability, leading to an underestimation of catastrophic and impoverishing health expenditures. Future research should explore the financial barriers that prevent households from accessing healthcare.

- ✔ Regional and Urban-Rural Disparities

While this study provided subnational analysis, future research could focus more deeply on regional and urban-rural disparities. A qualitative component could help uncover the contextual reasons for these disparities, including access to healthcare facilities and the effectiveness of health insurance programs.

- ✔ Longitudinal Analysis

Conducting longitudinal studies can provide a better understanding of trends over time and the long-term impacts of policy changes, such as health insurance expansion or fee waiver programs, on catastrophic and impoverishing health expenditures.

- ✔ Health System Reforms and Policy Evaluation

Future studies should evaluate the effectiveness of specific health system reforms, such as the community-based health insurance (CBHI) schemes or exemption services, in reducing financial hardship. This will provide actionable insights for scaling up successful interventions.

- ✔ Household Coping Mechanisms

Understanding how households cope with OOP expenditures (e.g., borrowing, selling assets, or forgoing treatment) could offer a deeper insight into the hidden financial and social impacts of healthcare costs.

- ✔ Focus on Vulnerable Populations

Future research should assess the impact of healthcare costs on particularly vulnerable groups, such as elderly individuals, people with chronic conditions, or low-income households, to tailor interventions effectively.

- ✔ Exploring Non-Monetary Barriers

In addition to financial aspects, future studies could examine other barriers, such as cultural, geographical, or social factors, that influence healthcare access and expenditure patterns.

- ✔ Integration of Advanced Statistical Techniques

Using advanced econometric and machine learning techniques can help identify and predict the determinants of catastrophic and impoverishing health expenditures with higher accuracy, allowing for more targeted policy interventions.

- ✔ International Comparisons

Comparative studies across countries with similar socio-economic contexts could help identify best practices and lessons learned from other regions dealing with catastrophic health expenditures.

By addressing these areas, future research can provide a more comprehensive understanding of financial hardship in healthcare and contribute to designing effective strategies to achieve Universal Health Coverage.

## Conclusion

As per the finding of the study catastrophic (1.49%) and impoverishing (0.83%) expenditure for health seems to be lower compared to sub Saharan African countries but there are still significant numbers of households facing it especially in Benishangul-Gumuz (4.12%), SNNP (3.76%) and Amhara (1.73%) regions. In addition household who has health insurance coverage, lower hospitalization and health service utilization rate has low incidence of incurring catastrophic health expenditure showing that out-of-pocket health payment is still an issue to be addressed in most regions of our country. It is also shown that catastrophic and impoverishing health expenditure is seen more in the poor than the rich ones resulting health inequality. So it is suggested that activities that reduce hospitalization rate, increase insurance coverage and addressing the poor must be in place so that the catastrophic health cost incurred can be lowered at national level.

## Data Availability

The data are available through the CSA web site: https://www.csa.gov.et/ or https://www.statsethiopia.gov.et/ or through the World bank micro data library website: https://microdata.worldbank.org/ and Users do not need to obtain the permission of the CSA to receive a copy of the data but will be asked to fill in a data access agreement.
